# Mesenchymal-Stromal Cell-like Melanoma-Associated Fibroblasts Increase IL-10 Production by Macrophages in a Cyclooxygenase/Indoleamine 2,3-Dioxygenase-Dependent Manner

**DOI:** 10.3390/cancers13246173

**Published:** 2021-12-07

**Authors:** Uğur Çakır, Anna Hajdara, Balázs Széky, Balázs Mayer, Sarolta Kárpáti, Éva Mezey, Pálma Silló, Gergely Szakács, András Füredi, Zoltán Pós, Barbara Érsek, Miklós Sárdy, Krisztián Németh

**Affiliations:** 1 Department of Dermatology, Venereology and Dermatooncology, Semmelweis University, 1085 Budapest, Hungary; cakir.ugur@semmelweis-univ.hu (U.Ç.); hajdara.anna@itk.ppke.hu (A.H.); szeky.balazs@itk.ppke.hu (B.S.); mayer.balazs@med.semmelweis-univ.hu (B.M.); skarpati@t-online.hu (S.K.); sillo.palma@med.semmelweis-univ.hu (P.S.); sardy.miklos@med.semmelweis-univ.hu (M.S.); 2 Károly Rácz Doctoral School of Clinical Medicine, Semmelweis University, 1085 Budapest, Hungary; 3 Roska Tamás Doctoral School of Sciences and Technology, Faculty of Information Technology and Bionics, Pázmány Péter Catholic University, 1083 Budapest, Hungary; 4 Adult Stem Cell Section, National Institutes of Dental and Craniofacial Research, National Institutes of Health, Bethesda, MD 20892, USA; mezeye@mail.nih.gov; 5 Drug Resistance Research Group, Institute of Enzymology, RCNS, Eötvös Lóránd Research Network, 1052 Budapest, Hungary; gergely.szakacs@meduniwien.ac.at (G.S.); furedi.andras@ttk.mta.hu (A.F.); 6 Department of Medicine I, Institute of Cancer Research, Comprehensive Cancer Center, Medical University of Vienna, 1090 Vienna, Austria; 7 Department of Genetics, Cell and Immunobiology, Semmelweis University, 1085 Budapest, Hungary; pos.zoltan@med.semmelweis-univ.hu (Z.P.); ersek.barbara@med.semmelweis-univ.hu (B.É.)

**Keywords:** melanoma-associated fibroblast, tumor-associated macrophages, IL-10, cyclooxygenase, indoleamine 2,3-dioxygenase

## Abstract

**Simple Summary:**

Melanoma is the deadliest form of skin cancer, and the number of newly diagnosed cases is on the rise. In recent years, it has become evident that melanoma-associated fibroblasts (MAFs), which surround the melanoma cells, play a key role in tumor growth and its ability to evade immune attack. We found that MAFs resemble bone marrow mesenchymal stromal cells (MSCs), and on the basis of this, we looked for effects that they might have on macrophages. Like MSCs, MAFs cause macrophages to produce IL-10, an anti-inflammatory agent. IL-10 contributes to cancer growth by suppressing natural anti-cancer immunity and can also interfere with anti-melanoma immunotherapies. Our findings may open new avenues for the development of anti-melanoma treatments based on MAF-macrophage interactions.

**Abstract:**

Melanoma-associated fibroblasts (MAFs) are integral parts of melanoma, providing a protective network for melanoma cells. The phenotypical and functional similarities between MAFs and mesenchymal stromal cells (MSCs) prompted us to investigate if, similarly to MSCs, MAFs are capable of modulating macrophage functions. Using immunohistochemistry, we showed that MAFs and macrophages are in intimate contact within the tumor stroma. We then demonstrated that MAFs indeed are potent inducers of IL-10 production in various macrophage types in vitro, and this process is greatly augmented by the presence of treatment-naïve and chemotherapy-treated melanoma cells. MAFs derived from thick melanomas appear to be more immunosuppressive than those cultured from thin melanomas. The IL-10 increasing effect is mediated, at least in part, by cyclooxygenase and indoleamine 2,3-dioxygenase. Our data indicate that MAF-induced IL-10 production in macrophages may contribute to melanoma aggressiveness, and targeting the cyclooxygenase and indoleamine 2,3-dioxygenase pathways may abolish MAF–macrophage interactions.

## 1. Introduction

MAFs are a melanoma-derived subtype of cancer-associated fibroblasts (CAFs) [1]. They are fibroblast-like cells that create a three-dimensional supporting scaffold around melanoma cells [2]. Their cancer-supporting function has been long described and it is similar to the role of mesenchymal stromal cells (MSCs) in supporting certain cell populations in the bone marrow [3]. MAFs produce cancer-protective molecules that augment melanoma growth, facilitate metastatic potential of primary melanoma cells, and may even assist melanoma cells in evading chemo- and/or immunotherapy [4,5]. MSCs protect bone marrow stem and progenitor cells by providing growth factors and nutrients and help to maintain an immune privileged milieu [6,7]. Just like MSCs, MAFs can modulate immune responses. When co-cultured in vitro, MAFs exert potent suppressive activity on NK cell-driven cytotoxicity and cytokine production [8,9]. Similarly, exposure to MAF-conditioned cell culture supernatants results in diminished CD8 lymphocyte functions, including decreased granzyme B expression, impaired killing activity, and an increase in negative immune checkpoint inhibitors such as TIGIT and BTLA [10]. In our previous work, we demonstrated the ability of MSCs to change the polarization of macrophages from a pro-inflammatory phenotype towards an anti-inflammatory character. This observation led to the discovery of various disease states, such as in sepsis, asthma, or sarcoidosis, where the immunomodulatory effect of MSCs may be beneficial [11,12,13]. The effect of MAFs on myeloid cells such as macrophages has been largely unexplored. In this present study, we wondered if MAFs are also able to influence the inflammatory properties of macrophages in their environment and if they possess stem cell properties such as MSCs.

## 2. Materials and Methods

Between 2015 and 2019, 32 stage-III/IV melanoma patients and 2 healthy blood donors were enrolled in our study, which was conducted at the Department of Dermatology, Venereology and Dermatooncology, Semmelweis University, Budapest, Hungary. After obtaining informed consent, blood specimens from healthy donors and freshly excised tumors from melanoma patients were collected and retrospectively analyzed as approved by the Hungarian Scientific and Research Ethics Committee of the Medical Research Council (ETT TUKEB; Decree No. 32/2007, supplements 32-2/2007 and 32-3/2007). The study was conducted in accordance with the ethical standards as dictated by the Declaration of Helsinki.

### 2.1. Cell Culture

The human monocytic cell line THP-1 (TIB-202), and BRAF mutated human malignant melanoma cell lines SK-MEL-28 (HTB72) and MALME-3M (HTB64) were purchased from American Type Culture Collection (ATCC; Rockville, MD, USA). THP-1 monocytes were cultured in Roswell Park Memorial Institute (RPMI) 1640 (Gibco™) medium supplemented with 10% fetal bovine serum (FBS) (Gibco™ Thermo Fisher Scientific, Inc., Waltham, MA, USA), 1% penicillin–streptomycin (P/S) (Gibco™), and 1% l-glutamine (Gibco™). BRAF mutated melanoma cells isolated from the excised tumors (MM-55) as well as SK-MEL-28 and MALME-3M were maintained in standard Dulbecco’s modified Eagle’s medium (DMEM) (Sigma-Aldrich, St. Louis, MO, USA) supplemented with 10% FBS, 1% penicillin–streptomycin (P/S), and 1% l-glutamine. MAFs were propagated in MAF medium (DMEM supplemented with 20% FBS, 1% penicillin–streptomycin (P/S) and 1% l-glutamine), and half of the medium was refreshed every other day.

### 2.2. MAF Isolation and Generation of MAF-Derived Conditioned Media

MAFs were isolated from either primary or metastatic tumors of melanoma patients and characterized as previously described [10]. First, the inner tumor mass was minced into ≈1 mm^3^ pieces and digested in 20 mL DMEM supplemented with 200 U/mL type IV collagenase and 0.6 U/mL dispase (Thermo Fisher Scientific, Waltham, MA, USA). MAFs were then separated from melanoma cells by utilizing a differential adhesion/trypsinization method. This protocol is based on the observation that fibroblasts such as MAFs adhere better to plastic than melanoma cells. In brief, the dispase/collagenase-digested tumor cell suspension was plated in a plastic cell culture dish. Then, 30 min later, floating cells were removed, and adherent cells were cultured (differential adhesion). Subconfluent cell cultures were trypsinized for 1 min, detached cells were removed, and still adherent cells enriched in MAFs were subcultured (differential trypsinization) [14]. Cultured MAFs were shown to be void of the melanoma markers melan-A and gp100 and positive for fibroblast-associated protein (FAP).

MAF cultures with 75–80% confluence were washed twice in phosphate-buffered saline (PBS) and further cultured in 10 mL basal medium (BM) consisting of DMEM, 1% P/S, 1% l-glutamine, and 0.5% BSA (Sigma-Aldrich). After 48 h, conditioned media (CM) derived from MAFs was collected.

### 2.3. Flow Cytometry Characterization of MAFs

Fluorescein isothiocyanate (FITC)-conjugated CD44, CD73, CD90, CD105, CD31, and CD45 (eBioscience, Thermo Fisher Scientific) cell surface markers in MAFs were analyzed by multicolor cytometry with Cytoflex V5-B5-R3 (Beckman Coulter, Brea, CA, USA) and FlowJo^®^ (Becton Dickinson and Company, Franklin Lakes, NJ, USA) software. Gating strategy consisted of eliminating 7AAD (eBioscience, Thermo Fisher Scientific) positive dead cells, and then FITC positive populations were compared to unstained control.

### 2.4. In Vitro Osteogenic Differentiation and Alizarin Red S Staining

For osteogenic differentiation, MAFs were seeded in 6-well tissue culture-treated plates and treated with DMEM containing 20% FBS, 1% P/S, 1% l-glutamine, 10 nM dexamethasone, 100 µM ascorbic acid, and 2 mM beta-glycerophosphate for 21 days. Media was changed every 3 days during the 21-day period of differentiation. To assess mineralization, we fixed cells in 4% paraformaldehyde for 15 min and stained them with Alizarin Red S (ARS) for 45 min. Excess dye was removed by washing the cells four times with double-distilled water.

### 2.5. In Vitro Adipogenic Differentiation and Oil Red O Staining

For adipogenic differentiation, MAFs were seeded in 6-well tissue culture-treated plates in low-glucose DMEM containing 20% FBS, 1% P/S, 1% l-glutamine, 0.5 mM 3-isobuthyl-2-methylxanthine (IBMX), 50 µM indomethacin, 0.5 µM hydrocortisone, 10 µM recombinant human insulin, and 10 µM troglitazone. Medium was changed every third day during the 21-day period of differentiation. To stain lipid droplets, we fixed cells in 4% paraformaldehyde for 10 min, washed them in PBS, and rinsed them in 60% isopropanol for 5 min. Then, we incubated cells in a 2:3 ratio of 3 mg/mL Oil Red O and double-distilled water for 15 min. Excess dye was removed by washing the cells four times with double-distilled water.

### 2.6. qRT-PCR Measurements of Osteogenic and Adipocenic Differantiated MAFs

Cell lysis and RNA extraction were performed using RNeasy mini-kit by Qiagen (Hilden, Germany). The M-MLV RT (Moloney Murine Leukemia Virus Reverse Transcriptase) enzyme (provided by Promega™) was used for cDNA-synthesis. qPCR was measured in a Roche Lightcycler^®^ 480 thermal-cycler. FAM-MGB-conjugated TaqMan Probes (by Thermo Fisher Scientific, Waltham, MA, USA) and *GAPDH* (assay ID: Hs99999905_m1, amplicon length: 122 bp) as a housekeeping control were used. For osteogenic differentiation, *ALPL* gene (assay ID: Hs01029144_m1) and *BGLAP* gene (assay ID: Hs01587814_g1) were measured at amplicon lengths of 79 and 138 bp, respectively. For adiopgenic differentiation, *PPARG* gene (assay ID: Hs01115513_m1) and *CEBPA* gene (assay ID: Hs00269972_s1) were measured at amplicon lengths of 90 and 77 bp, respectively.

### 2.7. Immunostaining of Melanoma Samples for FAP and Iba-1

After surgical excision, the tissue was fixed in buffered 10% paraformaldehyde and embedded in paraffin. Sections were cut onto positively charged slides at 6 µm thickness, baked overnight in a 65 °C oven, and were deparaffinized, and then antigen retrieval was performed in citrate buffer (pH 9) in a microwave oven. The sections were then blocked with BSA to avoid non-specific binding of the antibodies, and endogenous peroxidase activity was also blocked in order to not interfere with the staining procedure that followed. First, the tumor stroma was labelled using antibody to fibroblast activation protein (FAP) (ABCAM ab207178, rabbit monoclonal antibody) in 1:1000 dilution at 4 °C overnight, followed by 1 h incubation with a rabbit IgG VisUCyte HRP polymer (VC003 R&D Systems), and then an Alexa-594 conjugated Tyramide at 1:10,000 dilution. Following a second microwave session (to eliminate the primary antibody and inactivate the added HRP), the second primary antibody, Iba-1 (WAKO 019-19741), was applied to the sections at 1:2000 dilution, followed by the rabbit Visu-cyte polymer (R&D Systems, VC-003) and an Alexa-488 conjugated Tyramide (1:10,000 dilution). Finally, DAPI was used for nuclear staining. Negative controls included no primary antibody and/or no HRP conjugate. Visualization was performed with a Leica DMI6000 inverted fluorescence microscope using the LAX software [15].

### 2.8. Primary Monocyte Isolation

**Figure 1:**
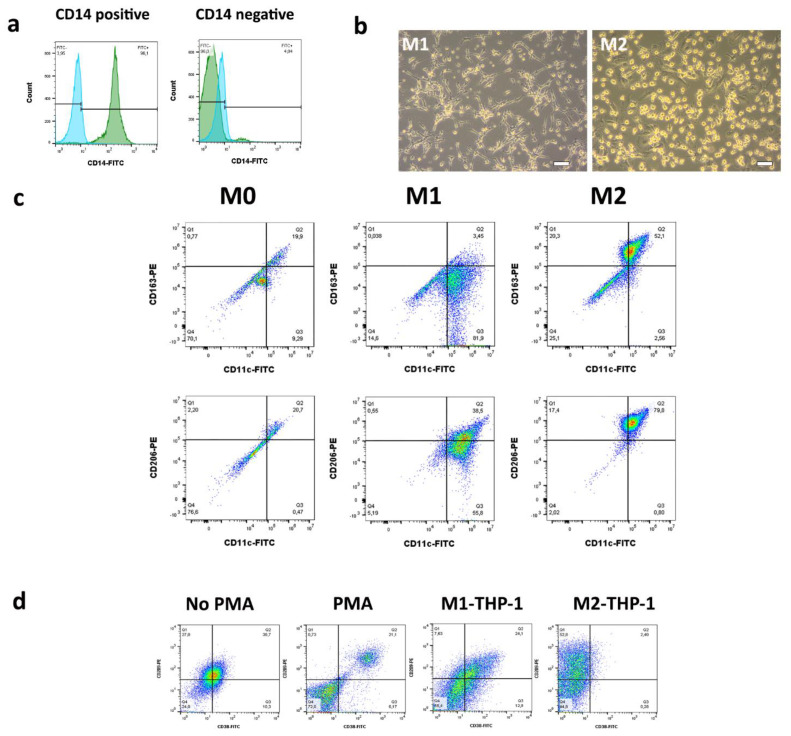
(**a**) CD14-positive (left) and -negative (right) PBMC fractions after magnetic bead separation. (**b**) Morphology of monocyte-derived M1 (left) and M2 (right) macrophages; scale bar = 20 µm. (**c**) CD163, CD206, CD11c cell surface marker expression in M0, M1, and M2-like macrophages. (**d**) CD38 and CD209 cell surface marker expression in non-differentiated THP-1 cells without PMA stimulation or PMA-stimulated and M1- and M2-differentiated THP-1 cells.

Monocytes were isolated from fresh peripheral blood mononuclear cells (PBMCs) of healthy individuals via Ficoll–Paque gradient centrifugation. CD14^+^ monocytes were isolated from PBMCs via positive selection using Miltenyi anti-human CD14 microbeads and an MS column (Miltenyi Biotec, Bergisch Gladbach, Germany) by magnetic activated cell sorting. The purity of the isolated cell population was confirmed by flow cytometry (BD FACSCalibur™ system, BD Biosciences, San Diego, CA, USA) using anti-human CD14 (FITC, Biolegend, San Diego, CA, USA) and following the manufacturer’s recommendations. Plots were analyzed with FlowJo software (Figure 1a).

### 2.9. M1/M2 Differentiation Assay

The CD14^+^ monocytes isolated from fresh PBMCs of healthy donors were differentiated into M1-like and M2-like macrophages in different cytokine milieu. The optimal concentration of cytokines and incubation times to achieve differentiation were determined by preliminary experiments. M1-like and M2-like macrophages were obtained following a 9-day incubation with 20 ng/mL granulocyte-macrophage colony-stimulating factor (GM-CSF), IFN-γ, LPS, IL-6, and 20 ng/mL macrophage colony-stimulating factor (M-CSF), IL-6, IL-13, and IL-4 cytokines, respectively. Cytokine-containing medium was refreshed on the fifth day.

M1-like macrophage morphology showed a roundish cell body and elongated cytoplasmic extensions, while M2-like macrophage cell morphology demonstrated roundish cell body and shorter, thicker cytoplasmic extensions after 9 days of differentiation (Figure 1b). M1-like CD11c [16,17] and M2-like CD206 [18,19] and CD163 [20] markers on macrophages were confirmed with flow cytometry Cytoflex V5-B5-R3 (Beckman Coulter, Brea, CA, USA) and FlowJo^®^ software (Figure 1c).

THP-1 monocytes were differentiated into macrophages of M0, M1, and M2-like phenotype, as described by Genin et al. [21]. First, THP-1 monocytes (2 × 10^5^ cells/well) were plated in 96-well plates and differentiated into M0-like macrophages by 24 h incubation with 20 ng/mL phorbol 12-myristate 13-acetate (PMA, Sigma-Aldrich), followed by 24 h incubation in fresh RPMI 1650 medium. M0-like macrophages were polarized into M1-like macrophages by 24 h incubation with 20 ng/mL of interferon-γ (IFN-γ) (R&D System) and 10 pg/mL of lipopolysaccharide (LPS) (Sigma-Aldrich). Macrophage M2-like polarization was achieved by 72 h incubation with 20 ng/mL of interleukin 4 (PeproTech) and 20 ng/mL of interleukin 13 (PeproTech). M1-like CD38 [22,23] and M2-like and CD209 [23,24] marker expression on macrophages was confirmed with flow cytometry (BD FACSCalibur™ system, BD Biosciences, San Diego, CA, USA). Plots were analyzed with FlowJo software (Figure 1d).

### 2.10. Cell Culture Assays

For MAF-macrophage co-culture assays, THP-1 monocytes (2 × 10^5^ cells/well) in 96-well plates were differentiated into macrophages of various phenotypes as described above. Following a PBS wash, 5 × 10^4^ MAF or pre-conditioned MAF cells (see below) per well were added and incubated in DMEM supplemented with 10% FBS, 1% penicillin–streptomycin, and 1% L-glutamine for 24 h. To enhance cytokine production, cells were stimulated with 1 µg/mL LPS for an additional 18 h. Lastly, the plates were centrifuged, and supernatants were collected and stored at −20 C. In case of co-culture of primary macrophages from healthy donors with MAF cells, this process was repeated but with 5 × 10^4^ cells per well (macrophages) and 25 × 10^3^ cells per well (MAFs).

For MAF titration (dose curve) assay, MAFs at 2 × 10^5^ cells per well with a twofold decreasing titration were added to a constant number of differentiated THP-1 macrophages at 2 × 10^5^ cells per well and incubated as described above.

For MAF monocultures, MAFs at 5 × 10^4^ cells per well were incubated in 96-well plates with DMEM supplemented with 10% FBS, 1% penicillin–streptomycin, and 1% L-glutamine for 24 h, followed by LPS treatment, as described previously.

### 2.11. Generation of Untreated and Chemotherapy or Small-Molecule Inhibitor-Treated Conditioned Media

Melanoma cell cultures reaching 75–80% confluence were washed twice in PBS and further cultured in 10 mL basal medium (BM) consisting of DMEM, 1% P/S, 1% l-glutamine, and 0.5% BSA (Sigma-Aldrich). After 48 h, media conditioned by cultured cells (conditioned media, CM) were collected. Twofold serial dilutions of CM in BM were made, and MAFs were incubated in diluted CM for 48 h. Subsequently, cells were washed in PBS. Preconditioned MAFs were used in co-culture assays as described above.

Melanoma tumor cells were treated with 1 of 5 drugs: 1 µM vemurafenib, 1 µM dabrafenib, 1 µM trametinib, 1 µM dabrafenib + 1 µM trametinib, or 500 µM dacarbazine (DTIC) for 48 h. These treatment concentrations were selected on the basis of previous cytotoxicity experiments and were demonstrated to be able to induce cell death in SK-MEL-28 and MALME3 melanoma cell lines. Subsequently, cells were washed in PBS and incubated in fresh culture medium for 48 h. The CM from chemotherapy treated cells was collected and MAFs were incubated in them for 48 h. These pre-conditioned MAFs were used in co-culture assays as described above.

### 2.12. Inhibitor Assay

NS-398 (selective COX2 inhibitor), SC-560 (selective COX1 inhibitor), 1-methyl-D-tryptophan (IDO inhibitor), and L-NG-Nitro arginine methyl ester (L-NAME; iNOS inhibitor) were tested in twofold dilution series starting with 8 µM, 8 µM, 8 mM, and 8 mM, respectively. These compounds were added at the initiation of the co-culture with MAFs and macrophages and incubated overnight before addition of LPS. Supernatants were assayed for IL-10 by ELISA after 18 h of LPS treatment.

### 2.13. ELISA

Supernatants from macrophage and MAF co-cultures were collected and measured by the R&D Systems IL-10 ELISA kit (Quantikine; R&D Systems, Minneapolis, MN, USA) according to the manufacturer’s instructions. Measurements were conducted in triplicate/quadruplicate. Absorbance was measured at 450 nm.

### 2.14. Statistical Analysis

We examined the differences between the groups for statistical significance by Student’s *t*-test or two-way ANOVA using Prism 7.0; Graphpad Software. A *p*-value of <0.05 was accepted as statistically significant. All experiments were performed in triplicate/quadruplicate.

## 3. Results

### 3.1. MAFs Expressed Traditionally Accepted MSC Markers and Were Also Able to Differentiate towards Osteogenic and Adipogenic Lineages

**Figure 2:**
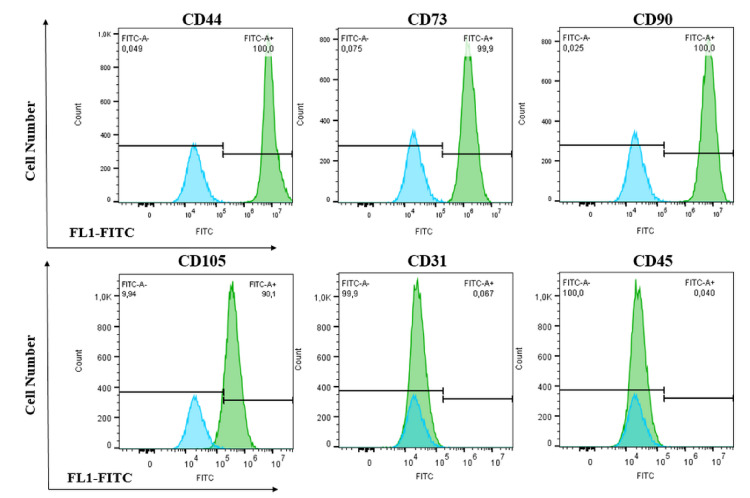
MSC-like marker expression of MAFs. Histograms show cell surface antigen expression of MAFs (green) compared to unstained control (blue), *n* = 3.

Considering the functional similarities between MAFs and MSCs, we wondered if MAFs express MSC surface antigens and if they are able to differentiate into mesodermal lineages. First, all cultured MAFs were shown to express the fibroblast marker FAP and to be void of melanoma markers such as melan A and gp100. Next, utilizing an array of MSC antibodies, we tested a select number of MAF batches (*n* = 3) and showed that close to 100% of MAFs express CD44, CD73, CD90, and CD105 antigens, previously selected by the International Stem Cell Society (ISCT) as part of the minimal criteria when defining MSCs [25]. MAFs did not express the endothelial marker CD31 or the hematopoetic marker CD45 (Figure 2).

**Figure 3:**
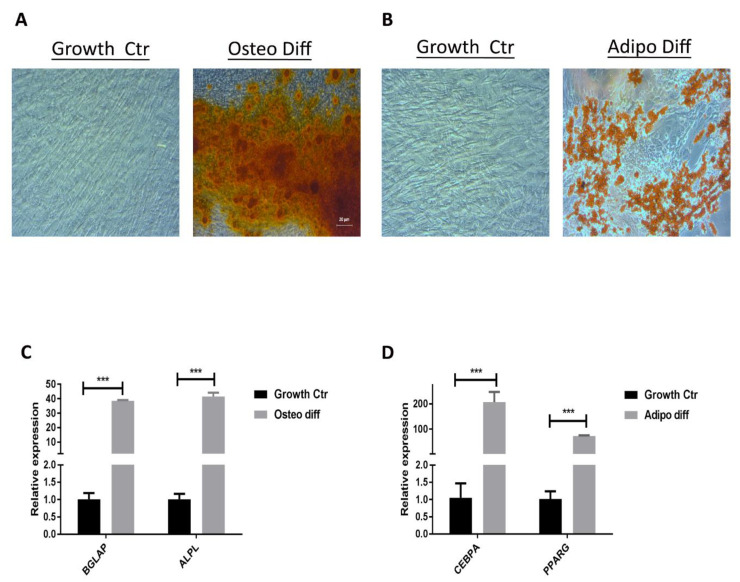
Differentiation of MAFs into osteocytes and adipocytes. (**A**) Alizarin Red S staining of calcium–phosphate complexes in MAFs treated with growth medium (left) and osteocyte differentiation medium (right). (**B**) Oil Red O staining of lipid droplets in MAFs treated with growth medium (left) and adipocyte differentiation medium (right). (**C**) Expression of osteogenic markers (ALPL, BGLAP) in undifferentiated MAFs and MAF-derived osteocytes (expression values relative to GAPDH expression). (**D**) Expression of adipogenic markers (CEBPA, PPARG) in undifferentiated MAFs and MAF-derived adipocytes (expression values relative to GAPDH expression). Error bars represent means ± s.e.m. *n* = 4. *** *p* < 0.01.

Subsequently, we showed that MAFs, just like MSCs, can differentiate into both adipocytes and osteoblasts in vitro. Upon stimulation with defined adipogenic and osteogenic differentiation cocktails, MAFs expressed the adipogenic markers CCAAT enhancer-binding protein alpha (CEBPA) and peroxisome proliferator-activated receptor gamma (PPARG) or osteogenic markers bone gamma-carboxyglutamate protein (BGLAP) and alkaline phosphatase (ALPL). In addition, MAFs were able to make oil droplets and form calcium deposits, as detected by Oil Red and Alizarin stains, respectively (Figure 3).

### 3.2. MAFs Were in Intimate Contact with Macrophages In Vivo

**Figure 4:**
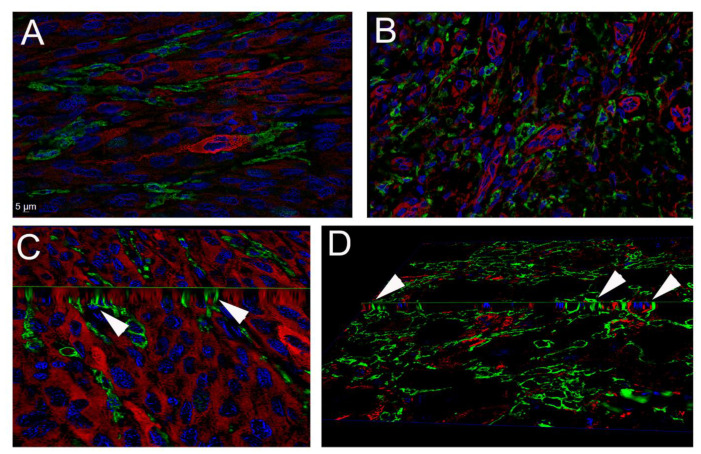
Immunohistochemistry of excised melanoma from two different patients. (**A**,**C**) Patient 1, (**B**,**D**) Patient 2. (**A**,**B**) The 0.5 µm thin optical sections from Z-stacks following deconvolution. (**C**,**D**) Images generated from slicing the three-dimensional Z stack; the arrows point at the intersection of the horizontal and vertical planes to demonstrate the very close connection between the membranes of Iba-1-positive macrophages (green fluorescence) and FAP-positive MAFs (red fluorescence). Blue fluorescence (DAPI) labels cell nuclei.

Previous studies have demonstrated that intravenously injected MSCs are eventually surrounded by recipient-derived macrophages, which facilitates the interactions between these two cell types [26]. Considering this observation, we wondered about the spatial distribution of MAFs and macrophages within the melanoma stroma. Two melanoma samples were examined with combined immunostainings. MAFs were identified with a commonly used cancer-associated fibroblast marker fibroblast activation protein (FAP), while macrophages were detected using ionized calcium binding adapter molecule 1, IBA-1 (also known as allograft inflammatory factor 1, AIF1), a highly specific marker used to detect tumor-associated macrophages [27,28] FAP-positive MAFs were readily identified within the cancer stroma, and interestingly, the majority of these stromal cells were surrounded by macrophages (Figure 4).

### 3.3. MAFs Increased IL-10 Secretion in THP-1 Cells and Primary Macrophages

**Figure 5:**
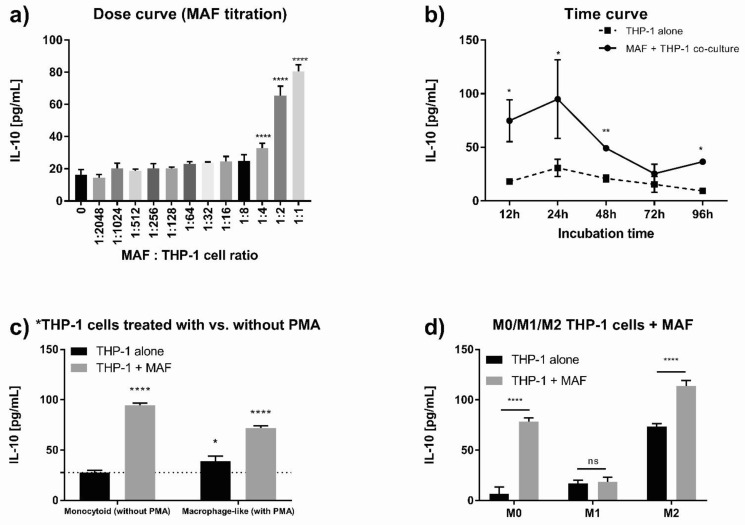
Effect of MAFs on IL-10 secretion in THP-1 macrophages. (**a**) IL-10 concentration of MAF-THP-1 co-cultures with a MAF/macrophage cell ratio between 1:2048 and 1:1, *n* = 4. (**b**) IL-10 concentration of THP-1 monoculture and MAF/THP-1 co-culture at 12 h, 24 h, 48 h, 72, and 96 h of incubation time, *n* = 4. (**c**) IL-10 concentration of monocytoid (without PMA pretreatment) and macrophage-like (with PMA pretreatment) THP-1 cells in monoculture and co-culture with MAFs, *n* = 6. (**d**) IL-10 concentration of M0-, M1-, and M2-like differentiated THP-1 macrophages in monoculture and co-culture with MAFs, *n* = 5. Error bars represent means ± s.e.m. * *p* < 0.05, ** *p* < 0.005, and **** *p* < 0.0001.

Because bone marrow stromal cells (BMSCs) are known to increase IL-10 secretion in monocytes/macrophages, we hypothesized that MAFs behave similarly. To examine this, we first co-cultured monocytoid THP-1 cells with MAFs in various ratios. While the number of THP-1 cells was kept constant, a gradual increase in the number of added MAFs resulted in a dose-dependent elevation of THP-1-derived IL-10 output, reaching an almost fourfold increase when equal number of THP-1 cells and MAFs were co-cultured (Figure 5a). Time curve analysis between 12 h and 96 h following LPS stimulation (36 h and 120 h total of co-culture time, respectively) demonstrated a peak stimulatory effect at 24 h (Figure 5b).

**Figure 6:**
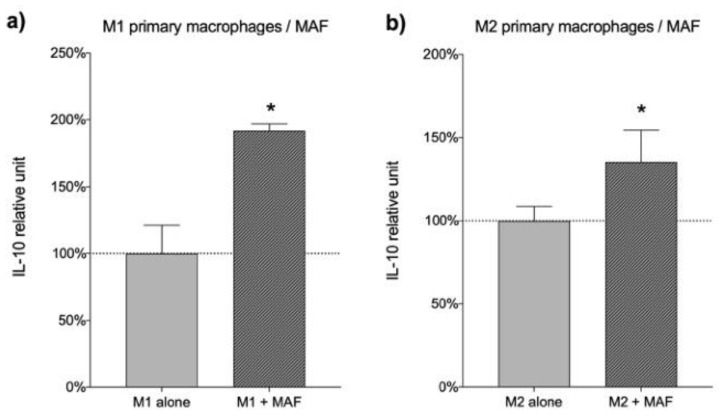
Effect of MAFs on IL-10 secretion in primary macrophages from healthy donors. Relative IL-10 concentration of MAF/M1-like (**a**) and M2-like (**b**) differentiated primary macrophage co-culture compared to monoculture, *n* = 3. Error bars represent means ± s.e.m. * *p* < 0.05.

To examine if MAFs can elicit IL-10 secretory response in various macrophage phenotypes, we pretreated monocytoid THP-1 cells with PMA or selected growth factors and co-cultured uncommitted M0, and polarized M1 or M2-like THP-1 macrophages with MAFs. M0 and M2 macrophages both responded with a robust increase in their IL-10 production, while M1 cells showed a slight, but not significant, increase in IL-10 secretion (Figure 5c,d). Subsequently, we repeated the co-culture experiments using primary monocyte-derived, in vitro-differentiated M1 and M2 macrophages, instead of the THP-1 cell line. In this case, the presence of MAFs resulted in a significant increase in IL-10 secretion in both M1 and M2 macrophages when compared to macrophage controls (Figure 6).

### 3.4. Thicker Melanomas Harbored More Immunosuppressive MAFs Compared to Thinner Tumors

**Figure 7:**
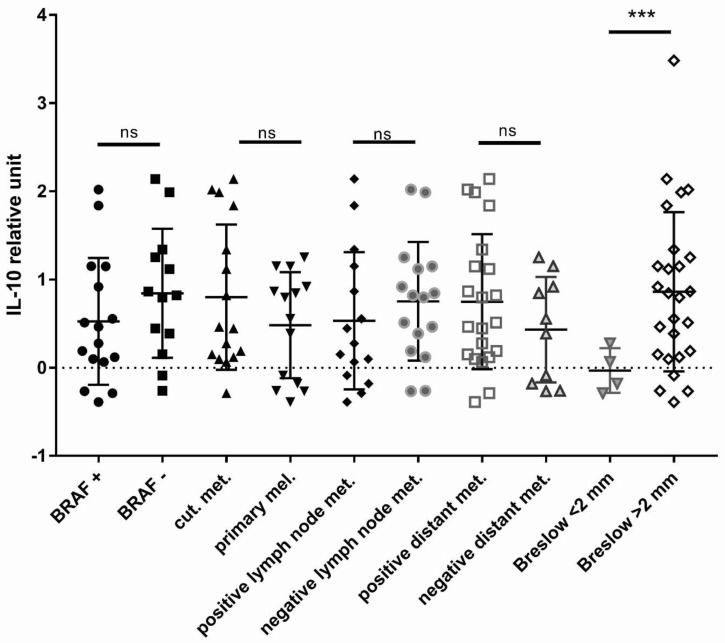
Clinical correlation of ex vivo IL-10 production of MAF macrophage co-cultures. Clinicopathological properties of melanomas and relative IL-10 concentration in supernatants of THP-1 macrophages co-cultured with MAFs isolated from tumors of various melanoma patients, *n* = 33 MAFs (isolated from 32 patients, MAFs from both primary tumor and metastasis of patient number 2 were isolated). ns: non-significant, *** p < 0.0005.

After establishing the boosting effect of MAFs on the IL-10 production of macrophages in vitro, we wondered if the degree of immunosuppression exhibited by MAFs may correlate with well-defined clinical parameters of melanoma patients (Table 1). First, we compared the IL-10-increasing ability of MAFs collected from primary melanoma samples of various Breslow depths. Interestingly, melanoma-derived MAFs from tumors thicker than 2 mm provoked a markedly higher IL-10 output in THP-1 macrophages as compared to thinner melanomas less than 2 mm deep (Figure 7). There was no difference between primary vs. metastatic melanoma-derived MAFs, and the BRAF status of the melanomas did not seem to influence the IL-10-increasing ability of MAFs either.

**Table 1:** Clinicopathological properties of MAF isolated patients and relative IL-10 concentration difference of MAF macrophage co-culture from macrophage monoculture. ALM: acral lentiginous melanoma, CM: cutaneous metastasis, DM: distant metastasis, F: female, LMM: lentigo maligna melanoma, LNM: lymph node metastasis, M: male, MI: mitosis index, NM: nodular melanoma, PT: primary tumor, SSM: superficial spreading melanoma, wt: wild-type.

Patient	MAF Origin	Gender	Age	Primary Melanoma Details					BRAF	LNM	DM	Relative IL-10 Change
				Subtype	Breslow (mm)	Clark	MI	Ulceration				
1	CM	M	90	unclassifiable	5.4	V	14	yes	wt	yes	yes	2.14
2	CM	F	79	SSM	2	IV			positive	yes	yes	1.84
	PT	F	79	SSM	2	IV			positive	yes	yes	−0.39
3	CM	F	80	NM	4	III		yes	wt	yes	yes	1.99
4	CM	M	73	SSM	0,87	II	6	yes	wt	yes	yes	0.82
5	CM	M	69	SSM	1	II			positive	yes	yes	−0.29
6	PT	M	84	LMM	5.15	IV	22	no	wt		yes	0.79
7	PT	F	76	NM	4.64	IV	15	no	positive	no	no	−0.27
8	CM	F	66	NM	9	V			positive	yes	yes	63.98
9	PT	M	23	unclassifiable	7.51	V	28	no	positive	yes		1.15
10	CM	M	70	NM	5.2	IV	18	yes	positive		yes	2.02
11	PT	F	50	SSM	2.92	IV	14	no	positive		yes	3.48
12	PT	M	56	SSM	1.77	III	4	no		yes		−0.18
13	PT	M	85	unclassifiable	10.26	IV	24	yes	positive	no		0.91
14	PT	M	74	NM	6.23	IV	18	yes	wt	yes		−0.09
15	CM	F	62	ALM	9.1	V	12	yes	positive		yes	0.19
16	CM	F	54	unclassifiable	18.21	V	42	yes	wt	yes	yes	0.15
17	CM	F	62	NM	9	IV			wt	yes	yes	1.34
18	CM	M	75	unclassifiable	3.34	IV	14	yes	wt	yes	yes	1.12
19	CM	M	72	unclassifiable	2.71	IV	18	no	positive	yes	yes	0.12
20	CM	F	52	SSM	10.58	V	28	no	positive		yes	0.51
21	CM	M	43	SSM	0.953	III	4	yes	positive	yes	yes	0.06
22	CM	F	82	Unknown primary					wt	yes	yes	0.45
23	PT	M	48	unclassifiable	17.5	V	26–29	yes	wt		yes	−0.26
24	PT	F	90	NM	13.24	IV	46	yes				0.85
25	CM	M	41	SSM	0.9	III	6	yes	positive	yes	yes	0.28
26	CM	M	67	SSM	6.18	V	5	yes	positive	yes	yes	0.01
27	PT	M	70	SSM	3.364	IV	3	yes	wt			0.39
28	PT	M	51	NM	5.17	IV	16	yes	wt	no	no	1.25
29	PT	M	81	SSM	5.336	IV	6–−8	yes	wt	yes	yes	0.87
30	PT	M	74	NM	13.24	V	48	yes	positive		yes	1.15
31	PT	M	57	unclassifiable	12.3	V	18	yes	positive	yes	yes	0.56
32	CM	F	71	SSM	3.4	IV	12	no	positive	yes	yes	0.46

### 3.5. Prior Exposure to Untreated or BRAF Inhibitor- or Chemotherapy-Treated Melanoma Cells Boosted IL-10-Increasing Ability of MAFs

**Figure 8:**
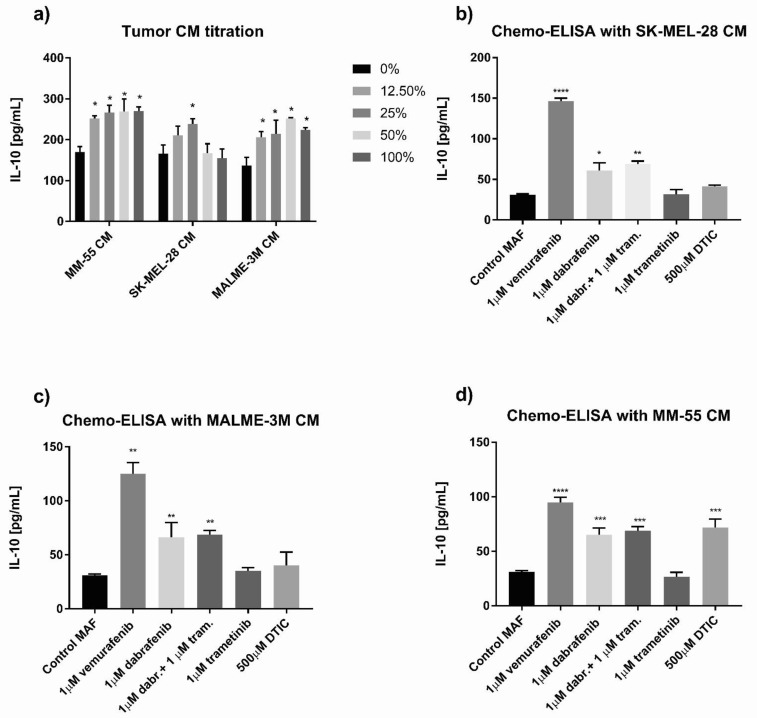
Preincubation of MAFs with conditioned media of melanoma cells. (**a**) IL-10 abundance in co-cultures of THP-1 macrophages with MAFs that were pre-incubated with different doses of conditioned media derived from MM-55, SK-MEL-28, and MALME-3M melanoma cells, *n* = 3. (**b**–**d**) IL-10 abundance in co-cultures of THP-1 macrophages with MAFs that were incubated with conditioned media from previously drug-treated MM-55, SK-MEL-28, and MALME-3M melanoma cells, *n* = 4. dabr. = dabrafenib, DTIC = dacarbazine, tram. = trametinib. Error bars represent means ± s.e.m. * *p* < 0.05, ** *p* < 0.005, *** *p* < 0.0005, and **** *p* < 0.0001.

We wondered if melanoma cells can influence how MAFs interact with macrophages. To test this, MAFs were incubated with increasing concentrations of conditioned media collected from either SK-MEL-28 or MALME-3 melanoma cell lines, or cultured, differential adhesion-selected primary melanoma cells. Such exposure to melanoma supernatants augmented the ability of MAFs to increase IL-10 production in THP-1 cells (Figure 8a). Interestingly, this effect was further facilitated when MAFs were cultured in the presence of BRAF inhibitor- or chemotherapy-treated cultured melanoma cells. When compared to untreated melanoma-conditioned MAFs, the small molecule inhibitors, vemurafenib, dabrafenib, and trametinib with dabrafenib, enhanced the ability of both melanoma cell lines and primary melanoma cells to stimulate MAFs, which ultimately led to an additional increase in THP-1-derived IL-10 secretion. Trametinib treatment of melanoma cells alone was unable to further potentiate the effect of MAFs on THP-1 cells. Finally, we treated melanoma cells with dacarbazine, an alkylating chemotherapeutic agent, and found that drug-treated primary melanoma cells magnified the IL-10 elevating effect of MAFs on THP-1 cells (Figure 8b–d).

### 3.6. Indoleamine 2,3-Dioxygenase (IDO) and the Cyclooxygenase (COX) Pathway Played a Critical Role in MAF-Driven IL-10 Increase

**Figure 9:**
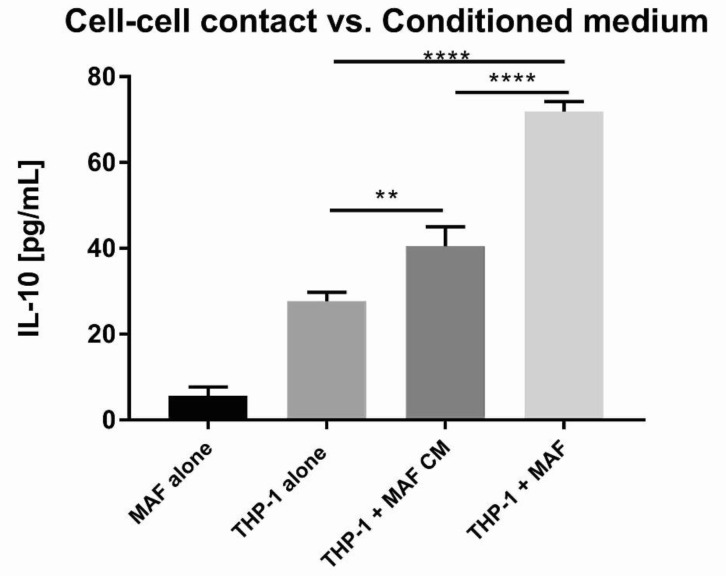
IL-10 concentration in supernatants of MAF monoculture, THP-1 monoculture, MAF-derived conditioned media (MAF CM)-treated THP-1 monoculture, and MAF/THP-1 co-culture, *n* = 5. Error bars represent means ± s.e.m. ** *p* < 0.001 and **** *p* < 0.0001.

**Figure 10:**
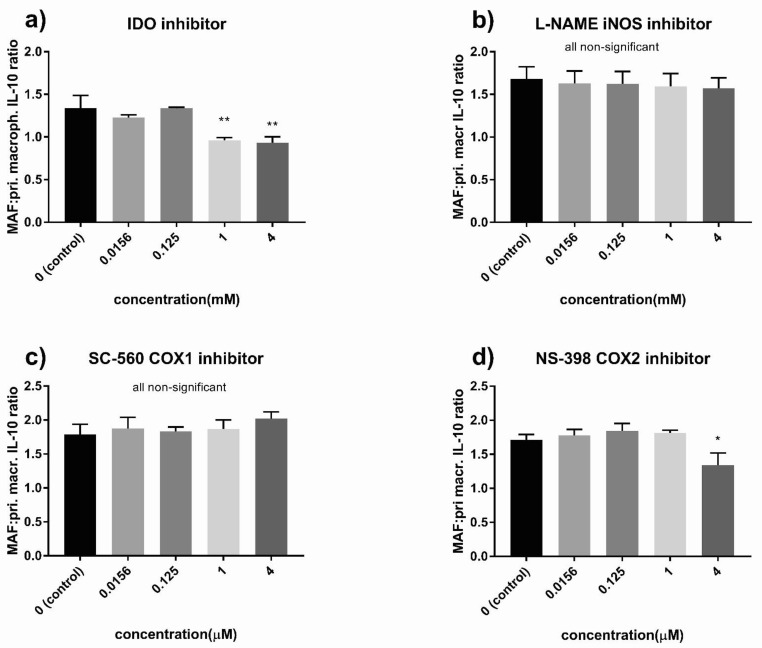
Inhibitors of IL-10 production in MAFs/primary macrophages co-culture. Ratio of IL-10 concentration of MAF/primary macrophage co-cultures to primary macrophage monocultures treated with different concentrations of 1-methyl-d-tryptophan (IDO inhibitor) (**a**), NG-nitro-L-arginine methyl ester (L-NAME) iNOS inhibitor (**b**), SC-560 COX1 inhibitor (**c**), and NS-398 COX2 inhibitor (**d**), *n* = 3. Error bars represent means ± s.e.m. * *p* < 0.05 and ** *p* < 0.005. pri. macr.: primary macrophage.

**Figure 11:**
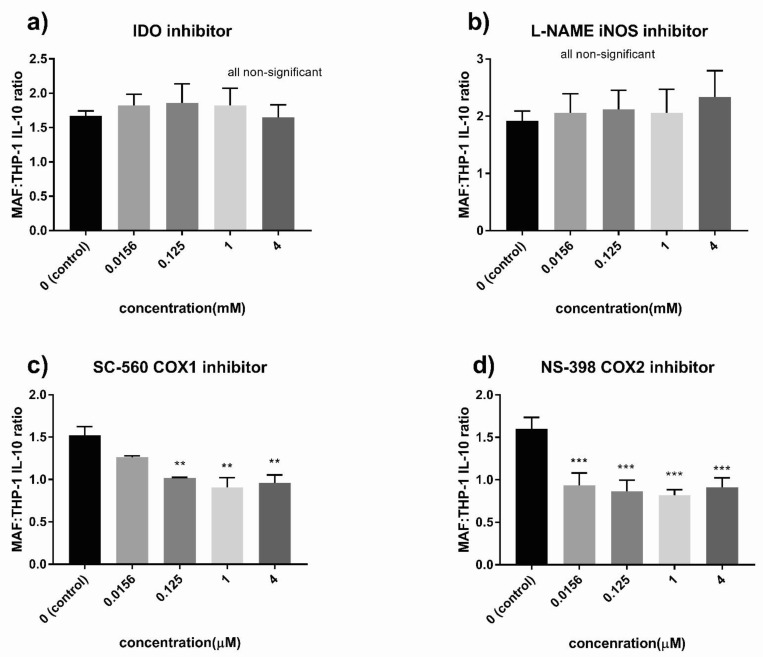
Inhibitors of IL-10 production in MAF/THP-1 macrophages co-culture. Ratio of IL-10 concentration of MAF/THP-1 co-cultures to THP-1 monocultures treated with different concentrations of 1-methyl-d-tryptophan (IDO inhibitor) (**a**), NG-nitro-L-arginine methyl ester (L-NAME) iNOS inhibitor (**b**), SC-560 COX1 inhibitor (**c**), and NS-398 COX2 inhibitor (**d**), *n* = 4. Error bars represent means ± s.e.m. ** *p* < 0.005 and *** *p* < 0.0005.

Finally, we set out to explore the molecular mechanisms involved in the immunosuppressive effect of MAFs. MAF monocultures on their own did not produce IL-10. To determine if cell–cell contact with macrophages is needed for the observed IL-10 stimulatory effect, we cultured THP-1 cells with MAFs with or without direct cellular contact. Although the observed IL-10-increase was greater in the direct co-culture setting, treatment of THP-1 cells with MAF-conditioned medium was able to increase IL-10 production as well, suggesting a role for soluble factors (Figure 9). Given the similarity between MSC-mediated and MAF-derived immunosuppression, we utilized selective pathway inhibitors known to interfere with MSC immunomodulatory effects. Inhibition of IDO led to a complete loss of IL-10 increase in primary macrophages (Figure 10a). In MAF-THP-1 co-cultures, inhibition of IDO effected both untreated and MAF-exposed macrophages, and therefore a co-culture-specific effect of IDO loss could not be observed (Figure 11a). Cyclooxigenase-1 inhibition abrogated IL-10 increase in THP-1 cells (Figure 11c), while COX2 inhibition diminished IL-10 production in both primary and THP-1 macrophages (Figure 10d and Figure 11d). iNOS inhibition had no effect on MAF-mediated IL-10 elevation (Figure 10b and Figure 11b, Table 2).

## 4. Discussion

In this study, we demonstrated that MAFs possess phenotypical and functional traits similar to bone marrow-derived MSCs, including potent immunoregulatory abilities when cultured with monocyte/macrophages.

MAFs are important elements of the melanoma microenvironment [29]. They are able to directly influence the growth and metastatic potential of melanoma cells, and mounting evidence suggests that they are also capable of modulating intra-tumoral immune responses by suppressing T cells and NK cells. In our present study, we demonstrated that MAF-exposed macrophages, just like MSC-treated myeloid cells, change character and increase their production of IL-10, the potent immunosuppressive cytokine.

The M1/M2 paradigm of macrophages was first described a long time ago [30]. M1 macrophages are believed to be pro-inflammatory, promoting anti-cancer immune responses, while M2 macrophages exhibit an immunosuppressive phenotype, dampening intra-tumoral inflammation and thus promoting evasion of anti-cancer immunity. Although the M1/M2 polarity and the corresponding cell surface markers and secreted molecules are well established, a homogenous population of these two phenotypic extremes is rarely seen in vivo. Rather, a heterogeneous mixture of macrophages is found in the tumor microenvironment, representing a continuum between M1 and M2 cells. Determining the net immunosuppressive effect of these macrophages is difficult, but the amount of select immunosuppressive molecules made by these cells may be suggestive of their role in evading anti-neoplastic immunity.

One such signature molecule is IL-10, which is considered to be one of the most potent immunosuppressive cytokines [31]. In fact, IL-10 production by tumor-associated macrophages in various cancers has been shown to correlate with disease progression and decreased survival [32,33]. Moreover, intratumoral IL-10 expression has been demonstrated to correspond with invasion depth and the metastatic potential of primary melanoma cells, while an increased serum level of IL-10 seems to render poor prognosis in advanced melanoma patients [34,35]. Therefore, we decided to study IL-10 secretion as our primary read-out of macrophage function in the presence of MAFs.

Our previous studies focused on BMSC macrophage interactions. When macrophages encounter BMSCs either in vivo or in vitro, they respond with decreased TNF-α production and an increase in IL-10 output [36]. Considering the similar phenotype of BMSCs and MAFs as demonstrated by our extensive immunophenotypical characterization and differentiation assays in the present work, we hypothesized that MAFs may have a similar effect on macrophages. To examine this, we decided to utilize a modified co-culture system that we previously developed to quantify the immunosuppressive potential of macrophages. The responder cells in this model can be either a macrophage cell line, such as THP-1 cells, or primary macrophages. THP-1 cells are readily available and easy to culture, providing a robust system to test our hypothesis, while data obtained from monocyte-derived primary macrophages are clinically more relevant. As expected, the presence of MAFs resulted in a marked increase in macrophage IL-10 secretion. This held true for both monocytoid and uncommitted macrophage-type THP-1 cells as well as M1- and M2-polarized THP-1 and primary macrophages. These data suggest that MAFs are capable of influencing all stages of macrophage development. CAFs secrete various chemokines such as MCP-1 and SDF-1 and are able to recruit monocytes to the tumor microenvironment [37]. Once in the cancer stroma, CAFs can directly interact with monocytes and instruct them to adopt a pro-tumorigenic, immunosuppressive phenotype, partly by inducing their IL-10 secretion. After these monocytes have committed to become tumor-associated macrophages, MAFs can continue to influence their behavior and promote IL-10 secretion in their unpolarized M0 and more committed M1 and M2 states as well.

One of the shortcomings of the above model is that MAF-macrophage interactions are studied outside of the context of melanoma. To address this issue, we repeated our experiments using MAFs previously exposed to melanoma. Prior exposure to primary or cell line-derived melanoma cells greatly promoted the MAFs IL-10 increasing ability. Interestingly, this immunosuppressive phenotype was further enhanced when MAFs were preconditioned with chemotherapy-treated melanoma cells. These observations imply that melanoma cells communicate with MAFs and facilitate their tumor-protective role in steady state and, even more so, under stress. The communication appears to be bidirectional. Once MAFs sense local danger signals and stress-induced melanoma molecules they can confer protection against chemotherapeutic agents and immune recognition via various mechanisms. These may include production of soluble factors such as HGF or neuregulin-1 that protect against chemotherapeutic drugs [38], or upregulation of programmed death ligands (PD-Ls) via the CXCL5/CXCR2 pathway that facilitate immune evasion [39]. Our results shed light on a possible new protective MAF-initiated pathway, governed by macrophage-derived IL-10. Once IL-10 is secreted, it has complex effect on cancer growth. It has been shown to directly support melanoma proliferation, stimulate angiogenesis, and suppress anti-tumor immune responses [40]. Another study using in vitro three-dimensional reconstructed organotypic human melanoma-in-skin model with melanoma cell line cells, healthy donor-derived epidermal cells, and fibroblasts demonstrated an increase of IL-10 mRNA production in all cells and a IL-10-dependent M2-like differentiation of monocytes [41]. Our study focused on patient-derived MAFs, and although MAFs did not produce IL-10 in monoculture, they induced a robust IL-10 production of macrophages, which could be increased by preconditioning of MAFs with tumor-derived conditioned media.

The degree of immunosuppression exerted by MAFs may differ greatly in individual patients. Capturing these differences is challenging, but our ex vivo co-culture system may offer a possible tool to predict the immunosuppressive ability of these cells. Our preliminary data show that MAFs derived from thicker melanomas are more immunosuppressive than MAFs obtained from thinner melanomas. This observation is in line with other studies demonstrating increased overall IL-10 expression in thicker melanomas [42,43]. Although our findings are limited by the small number of cases we could examine, if validated by larger studies, our assay may serve as an ex vivo tool to measure the immunosuppressive capacity of MAFs in patients. This could ultimately help predict disease prognosis and potential response to various targeted molecular and immunomodulatory treatments.

The communication between various stromal fibroblast types such as BMSCs and immune cells—mostly T lymphocytes—has been studied extensively. There are several molecular pathways that have been proposed to play an important role in mediating these interactions. The role of cyclooxigenase and nitric oxide pathway has been implicated in BMSC lymphocyte/macrophage interactions in murine models, while the IDO pathway was found to be critical in human BMSC/lymphocyte interactions [44,45,46,47]. Similarly, the same molecules have been implicated before in orchestrating a cancer-supportive microenvironment [48,49].

The COX1 and COX2 enzymes are both capable of making prostaglandins such as PGE2, PGF2, or prostacyclines [50]. COX1 is expressed ubiquitously, while the expression of COX2 is inducible under inflammatory conditions or in cancers [51]. The role of COX2 in melanoma has been suggested by various studies. COX2 expression in melanoma cells seems to correlate with invasion depth, and the role of COX2 has been also implicated in tumor angiogenesis, BRAF resistance, and immune evasion during check-point inhibitor therapy [52].

IDO is another key immunoregulatory molecule expressed in melanoma [53]. Its enzymatic function converts the amino acid tryptophane into kynurenin, which in turn inhibits cytotoxic CD8 T cells and NK cells and helps recruit immunosuppressive regulatory T cells and myeloid-derived suppressor cells into the tumor microenvironment [54]. Intriguingly, it has been recently shown that PGE2 drives the expression of IDO in human melanoma cells, and inhibition of COX2 results in immune destruction of IDO-expressing tumor cells [55].

Last but not least, the iNOS pathway has been recently reported to support melanoma growth via the upregulation of the oncogenic PI3K-AKT pathway, and increased intratumoral iNOS activity has also been linked to poor outcomes in melanoma patients [56,57].

In this study, we interrogated all three above pathways and found that intact function of both cyclo-oxygenases and IDO are critical in the immunomodulatory effect elicited by MAFs. Blocking the iNOS pathway, on the other hand, seemed to have no bearing on the MAF-mediated IL-10 increase. Although the idea to target CAFs has been around for decades, CAF-specific therapies have not yet led to a breakthrough. This is mainly because there are too many similarities between normal tissue fibroblasts residing in various organs and CAFs, recruited by cancers. An alternative approach could be to identify molecular pathways that are involved in multiple oncogenic processes, including cancer proliferation, angiogenesis, and CAF-mediated support of cancer cells. The more mechanisms we find that depend on a certain unique molecular pathway, the higher the likelihood that antagonizing this master regulatory pathway may be therapeutic as a monotherapy or together with other targeted molecular or immunomodulatory treatments. Our data add an important piece to the puzzle of the complex picture of melanoma biology. The fact that MAF/macrophage interactions are driven by both the cyclooxygenase pathway and IDO may boost the efforts to repurpose already existing COX inhibitors and develop novel IDO inhibitors to treat melanoma patients.

## 5. Conclusions

MAFs were shown to possess stem cell properties and to play an important role in regulating macrophage functions, promoting a pro-tumorigenic, IL-10-rich environment. On the basis of these observations, we believe that assaying minimally cultured MAFs in the presence of macrophages may help us better understand the role of stromal microenvironment in fostering tumor-immune privilege, and new data can ultimately lead to the development of novel prognostic tools and innovative therapies.

## Figures and Tables

**Table 2:** Summary of IL-10 inhibition in either MAF + THP-1 macrophage co-culture or MAF + primary macrophage co-culture. “Inhibited” indicates that IL-10 production of co-culture was inhibited by the effect of inhibitor, while “not inhibited” refers to lack of inhibition.

Co-culture	IDO Inhibitor	L-NAME iNOS Inhibitor	SC-560 COX-1 Inhibitor	NS-398 COX-2 Inhibitor
**MAF + THP-1**	not inhibited	not inhibited	inhibited	inhibited
**MAF + primary macrophage**	inhibited	not inhibited	not inhibited	inhibited

## Data Availability

All data generated or analyzed during this study are included in this published article. Further data are available on reasonable request from the corresponding author.
